# Systematic Control of Anodic Aluminum Oxide Nanostructures for Enhancing the Superhydrophobicity of 5052 Aluminum Alloy

**DOI:** 10.3390/ma12193231

**Published:** 2019-10-02

**Authors:** Chanyoung Jeong, Hyejeong Ji

**Affiliations:** Department of Advanced Materials Engineering, Dong-eui University, 176 Eomgwang-ro, Busanjin-gu, Busan 47340, Korea

**Keywords:** aluminum alloy, multistep anodization, pore-widening, pillar nanostructure, superhydrophobic surface

## Abstract

The recent increased interest in the various applications of superhydrophobic surfaces necessitates investigating ways of how this property can be enhanced further. Thus, this study investigated how superhydrophobic properties can be enhanced through the formation of anodic alumina nanostructures on 5052 aluminum alloy. A multistep anodizing process that alternates two different anodizing modes, mild anodization (MA) and hard anodization (HA), with an intermediate pore-widening (PW) process was employed. Multistep anodization was employed in two different ways: an MA → PW → HA process and an HA → PW → MA process. Both routes were conducted with PW durations of 40, 50, and 60 min. The well-defined nanostructures were coated with a self-assembled monolayer (SAM) of FDTS (1H, 1H, 2H, 2H-perfluorodecyltrichlorosilane). The contact angle values of water droplets were maximized in the pillar-like nanostructures, as they have a less solid fraction than porous nanostructures. With this, the study demonstrated the formation mechanism of both nanoscale pillar and nanoscale hierarchical structures, the wettability of the superhydrophobic surfaces, and the relationship between PW duration time with wettability and the solid fraction of the superhydrophobic surfaces.

## 1. Introduction

As superhydrophobic surfaces have high static contact angles (CA) greater than 150° with water droplets, they have recently attracted much interest in surface science due to their utility in a range of applications, including self-cleaning, anti-icing, oil–water separation, and anticorrosion processes [1,2,3,4,5,6,7,8,9,10,11,12,13]. To create such a surface for these various applications, fabrication techniques for creating micro- and/or nanostructures on surfaces coated with low surface-energy materials must be developed. In recent years, numerous methods have been used to design and fabricate superhydrophobic surfaces for technologies, including solar cells, gas sensors, and biomedical applications [14,15,16].

As the most abundant metal in the earth, aluminum (Al) and its alloys have been widely used in structural engineering—automotive, aerospace, building, shipment, and machine industries—due to their good thermal and electrical conductivity, low density, excellent strength, and design parameters. However, aluminum alloys have low corrosion resistance that requires surface treatment application [17,18,19]. Therefore, it is of great scientific significance and industrial value to be able to fabricate functional superhydrophobic surfaces on aluminum alloys.

To construct superhydrophobic surfaces, two kinds of approaches have been suggested: fabricating a hierarchical structure and modifying surface roughness with low surface energy materials [2,16,20]. The implementation of a superhydrophobic surface on the aluminum surface depends on controlling the surface shape modification and self-assembled monolayer coating. Among the known surface treatment methods, electrochemical anodization processes have been particularly convenient for modifying the surface shape through aluminum alloy surface etching. It is generally known that anodic aluminum oxide (AAO) films consist of hexagonal nanostructures arranged in cylindrical pillars. The nanopore structure of AAO films is defined by the pore diameter (D_p_) and interpore distance (D_int_), which can adjust the surface shape by modulating the anodization voltage [2,21,22,23,24,25]. The D_p_ of these films can be increased through pore widening, which consists of immersing the sample in a phosphoric acid solution after anodization. There is a similar study that used two-step electrochemical processes to achieve a similar effect of wetting properties on stainless steel surfaces, albeit on stainless steel surfaces [26]. Despite the similarity, the different composition of stainless steel and 5052 aluminum alloy are different, allowing different nanostructures to be shaped during the anodization process.

In this work, we focus on fabricating hierarchical nanostructures on Al 5052 substrates by employing multistep anodization with an intermediate pore-widening (PW) step. Modulating the anodization voltage between mild anodization (MA) at 40 V and hard anodization (HA) at 80 V, as well as varying the PW duration, can provide optimized superhydrophobic properties. We also investigated the superhydrophobic properties of the hierarchical structure of AAO modified with a self-assembled monolayer coating.

## 2. Materials and Methods

Aluminum alloy plates (Al 5052), measuring 10 mm × 30 mm and having 1-mm thickness, were used as substrates. Prior to anodization, the substrates were ultrasonically degreased in an acetone and ethanol mixture and finally rinsed with deionized water. They were subsequently electropolished in a perchloric acid and ethanol mixture (1:4 volumetric ratio) under an applied potential of 20 V for 60 s to remove irregularities on the surface. The polished aluminum alloy was used for the working electrode (anode), and a platinum electrode was employed as the counter electrode (cathode). The two electrodes were separated by a distance of 5 cm. The first anodization was conducted in a 0.3-M oxalic acid solution at 0 °C for 6 h. The anodic aluminum oxide (AAO) layer formed by the first anodization step was removed by soaking in an aqueous mixture solution of 1.8 wt. % chromic acid and 6 wt. % phosphoric acid at 65 °C for 10 h [27,28]. Then, the same conditions as used in the first anodizing step were applied to the second anodizing step. The second anodizing step involved both the mild anodization (MA) and hard anodization (HA) modes. The MA mode was applied at 40 V for 30 min, and the HA mode was applied at 80 V for 30 s [24]. Anodization was carried out in a constant voltage using a DC power supply with the size of aluminum dipping into the electrolyte. To make the pores wider, the AAO layers were dissolved in 0.1 M phosphoric acid at 30 °C for various immersion times: 40, 50, and 60 min. The same conditions used in the second anodizing step were applied to a third anodizing step to attempt the fabrication of hierarchical nanostructures (see Table 1).

To make a superhydrophobic AAO surface, the fabricated AAO surfaces were coated with a self-assembled monolayer (SAM) of FDTS (1H, 1H, 2H, 2H-perfluorodecyltrichlorosilane). Before coating, the samples were cleaned with O_2_ plasma for 15 min to remove organic residues and make the surfaces hydrophilic, and were then dried in the air and baked at 150 °C for 10 min. Then, the samples were coated with FDTS for 24 h in vacuum. For the examination of surface hydrophobicity after SAM coating, static contact angles were measured with a contact angle meter at room temperature. All the measurements were performed at five positions on each sample with a sessile droplet (~3.5 µL) of deionized water. The surface morphology of the samples was observed by a field emission scanning electron microscope (FE-SEM).

## 3. Results and Discussion

### 3.1. Fabrication Approach

**Figure 1:**
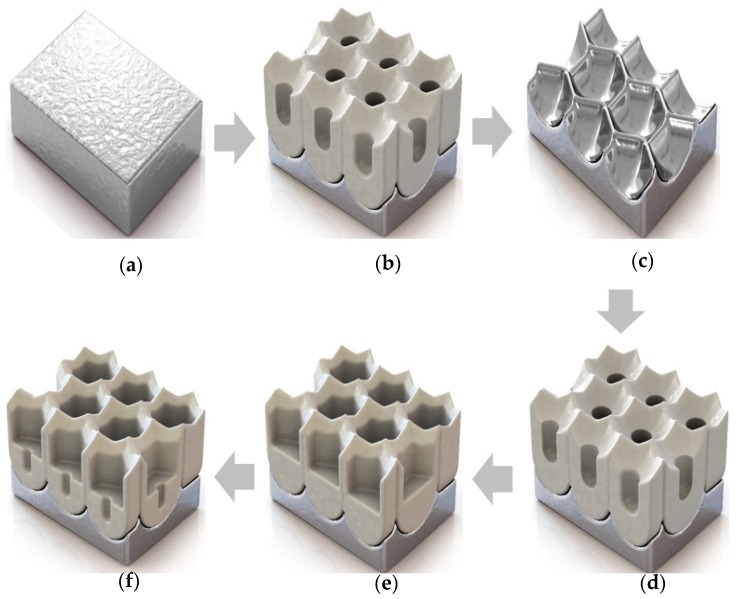
Schematic of the fabrication of a hierarchical anodic aluminum oxide (AAO) structure through multistep anodization; (**a**) Electropolished aluminum 5052; (**b**) First anodization; (**c**) Remove anodic aluminum oxide; (**d**) Second anodization; (**e**) Pore-widening; and (**f**) Third anodization.

Anodization has been increasingly used for making various porous nanostructures on aluminum substrates [29,30,31]. Control of the pore parameters, comprised of D_p_ and D_int_, is achieved by modulating anodization conditions such as voltage, temperature, and concentration. Anodization voltage, in particular, is the main determinant of D_p_ and D_int_. For example, compared to the MA mode, the HA mode uses relatively high applied voltage during anodization, which creates larger pore sizes [24]. In addition, if a PW step is performed with anodization, the range of pore sizes that can be obtained is extended. Figure 1 schematically shows the fabrication procedure for hierarchical structures using multistep anodization.

First, an Al 5052 sheet is electropolished to obtain a smooth surface. The electropolished Al 5052 is anodized in oxalic acid to fabricate an initial nanoporous alumina layer in the first anodization. Then, this nanopore layer is removed by chemical etching for the second anodization, in which the alumina is anodized again to fabricate a new AAO film under different anodization conditions (i.e., MA and HA steps). Then, this porous nanostructure is widened in phosphoric solution with different durations. Finally, in the third anodization, porous alumina is anodized to fabricate a distinctly different structure using the same conditions as the second anodization step. The different values of D_p_ and D_int_ that result from variations in the anodization voltage applied during the second and third anodization steps and during the pore-widening (PW) duration employed between these steps produce a hierarchical structure.

### 3.2. Effect of the Voltage Morphology

**Figure 2:**
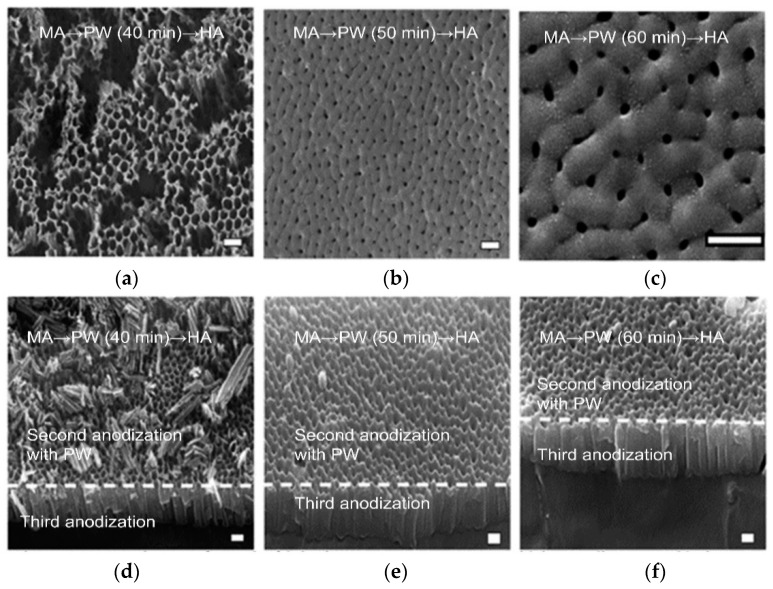
FE-SEM images of AAO for the MA → PW → HA fabrication step with an intermediate pore-widening duration of (**a**) to (**c**) (**a**,**d**) 40 min (Sample A), (**b**,**e**) 50 min (Sample B), and (**c**,**f**) 60 min (Sample C). Figures (**a**) to (**c**) shows the top view, while (**d**) to (**f**) shows the cross-section. Scale bar = 200 nm.

**Figure 3:**
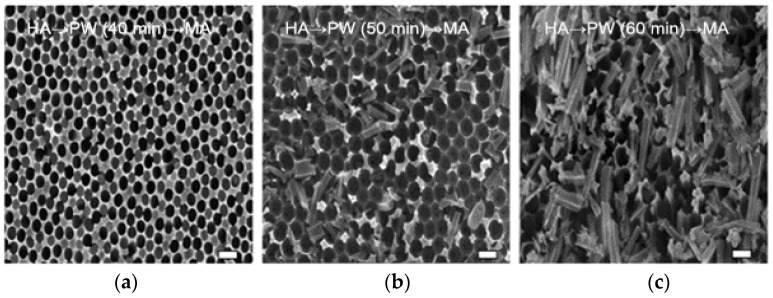

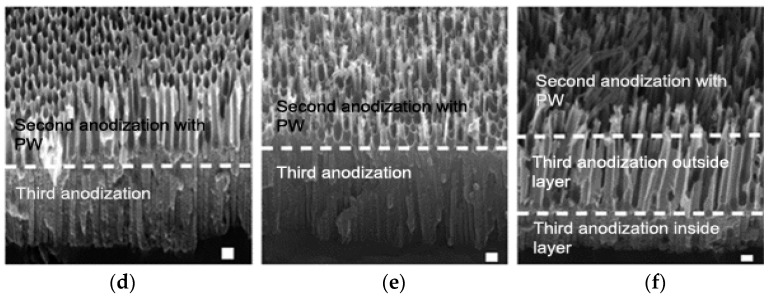
FE-SEM images of AAO for the fabricating the HA → PW → MA step with an intermediate pore-widening duration of (**a**,**d**) 40 min (Sample D), (**b**,**e**) 50 min (Sample E), and (**c**,**f**) 60 min (Sample F). **a**–**c** shows the top view, while **d**–**f** shows the cross-section. Scale bar = 200 nm.

The study investigated the effect of anodization voltage under a fixed PW time (40 min). FE-SEM images of the electropolished Al 5052 surfaces were subjected to either an MA → PW → HA process or an HA → PW → MA process, as shown in Figure 2 and Figure 3, respectively.

Different pore diameters and interpore distances derived from the applied anodization voltage employed were observed depending on whether the MA or HA step was applied first. Table 2 presents the average values of D_p_ and D_int_. The values of D_p_ and D_int_ were investigated in the cross-sectional images.

For the samples fabricated using either the MA → PW → HA or the HA → PW → MA process, the anodization and PW parameters (i.e., electrolyte used, temperature, and concentration) are constant. Figure 2d shows the FE-SEM image of the AAO after MA → PW → HA anodization. The MA of the second anodization resulted in D_p_ = 85 and D_int_ = 100. After a 40-min PW step, the HA of the third anodization resulted in D_p_ = 30 nm and D_int_ = 115 nm (Sample A). The structural morphology created by the 40-min PW process for dissolving the cell wall exhibited a pillar structure consisting of a combination of collapsing and individually standing pillars. Figure 3d shows the FE-SEM image of the AAO that underwent the reverse process: HA → PW → MA. The HA results of the second anodization were D_p_ = 95 nm and D_int_ = 137 nm. After the PW step, the MA results of the third anodization were D_p_ = 19 nm and D_int_ = 94 nm (Sample D). The D_p_ and D_int_ values for the MA → PW → HA process were greater than those resulting from the HA → PW → MA process. The results suggest that hierarchical structures can be fabricated by modulating the anodization voltages, and that the PW step influences both D_p_ and D_int_ of the second anodization region, but not of the third anodization region. It is generally accepted that the D_p_ and D_int_ of a porous nanostructure can be adjusted by anodization conditions such as the electrolyte type, anodization voltage, current density, and temperature. According to the following equations, D_p_ and D_int_ are dependent on the anodization voltage [32,33]:

(1) $$D_{P}=\lambda_{P}\times U,$$

(2) $$D_{int}=\lambda_{int}\times U$$

where D_p_ is the pore diameter (nm), λ_p_ is a proportionality constant approximately equal to 1.29 nm/V, D_int_ is the interpore distance (nm), λ_int_ is a proportionality constant approximately equal to 2.5 nm/V, and U denotes an anodization voltage. From these relationships, it is apparent that a higher applied voltage results in greater values for both D_p_ and D_int_. However, it can be confirmed in this case that the values of D_p_ and D_int_ are both small. Aluminum ions from the aluminum alloy dissolve and form AAO after oxidation during anodization [34]. It is expected that impurities in 5052 aluminum alloy will limit the oxidation reaction during anodization. Despite the same HA conditions, the D_p_ resulting from HA used in the second anodization is greater than the D_p_ resulting from HA used in the third anodization, as shown in Table 2. This difference is due to the influence of the PW step after the second anodization. Therefore, a porous hierarchical nanostructure can be fabricated with various sizes of D_p_ and D_int_ and different shapes by employing an anodization voltage between the MA and HA steps.

### 3.3. Effect of Pore-Widening Time on Morphology

This study investigated the impact of PW duration under alternative anodization processes such as multistep MA and HA. Figure 2 shows the FE-SEM images of the MA → PW → HA sequence fabricated by controlling the two types of anodization voltage and the intermediate different PW duration. Subsequently, FE-SEM images of the HA → PW → MA processes through stepwise anodization display the effects of the MA and HA sequences and processes in the intermediate PW step (see Figure 3). To study the effect of the PW duration, the PW time was varied from 40 min to 60 min. Table 2 shows the average values of D_p_ and D_int_ for each anodization sequence. For the samples with MA → PW → HA and HA → PW → MA, the anodization parameters, temperature, electrolyte, and concentration are constant. In the case of the MA → PW → HA process, a PW time of 40 min exhibits a hybrid structure in the second anodization region. In particular, the AAO structure of the second anodization region completely disappeared after a 40 min PW duration. Furthermore, a 40 min PW did not cause a structural change in the third anodization region. PW durations of 50 and 60 min resulted in D_p_ = 31 nm and 33 nm, respectively, and D_int_ = 182 and 219 nm, respectively, in the third anodization region. Therefore, as the PW time increases, D_p_ and D_int_ increase during the third anodization as a result of the AAO dissolution from the second anodization region. It can be seen that PW in phosphoric acid accelerates the dissolution of AAO during the third anodization for the MA → PW → HA process (samples A, B, and C).

In the case of the HA → PW → MA process, the values of D_p_ in the second anodization region are 95 nm and 134 nm as the PW time increases from 40 min to 50 min, respectively. When the PW time continuously increases to 60 min, it was observed that the structure in the second anodization region was etched away. D_int_ in the second anodization region shows a similar trend. On the other hand, D_p_ and D_int_ in the third anodization region did not show significant differences for PW times of 40 min and 50 min. However, a 60 min PW time resulted in a hierarchical structure during the third anodization. The upper bounds of D_p_ and D_int_ in the third anodization region were found to be 135 nm and 182 nm, respectively. The lower bounds of D_p_ and D_int_ in the third anodization region were found to be 16 nm and 99 nm, respectively (Samples D, E, and F). The AAO outer layer grew during the second anodization, while the AAO inner layer close to the substrate grew during the third anodization [23].

### 3.4. Effect of Multistep Anodization on Wettability

The wettability of the samples was assessed by measuring the static contact angle (CA) (see Table 3).

**Figure 4:**
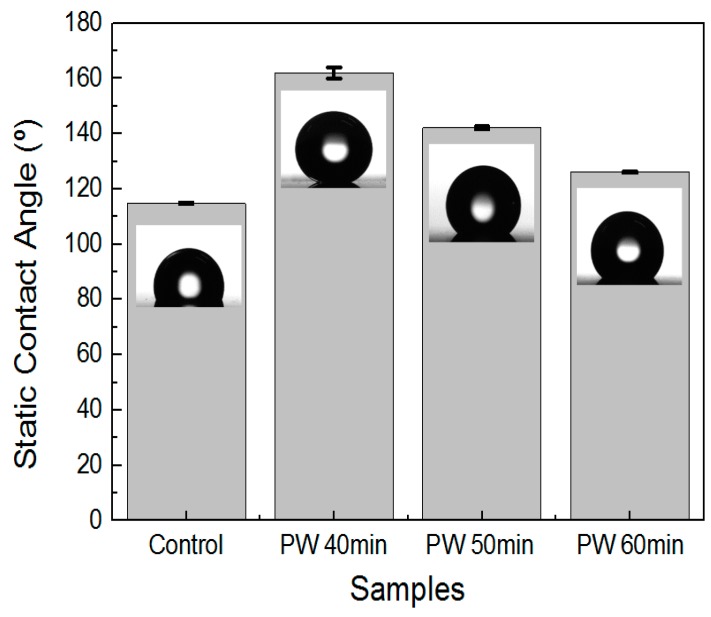
Contact angle measurements on the AAO fabricated with the MA → PW → HA process with different PW durations and coated with FTDS.

**Figure 5:**
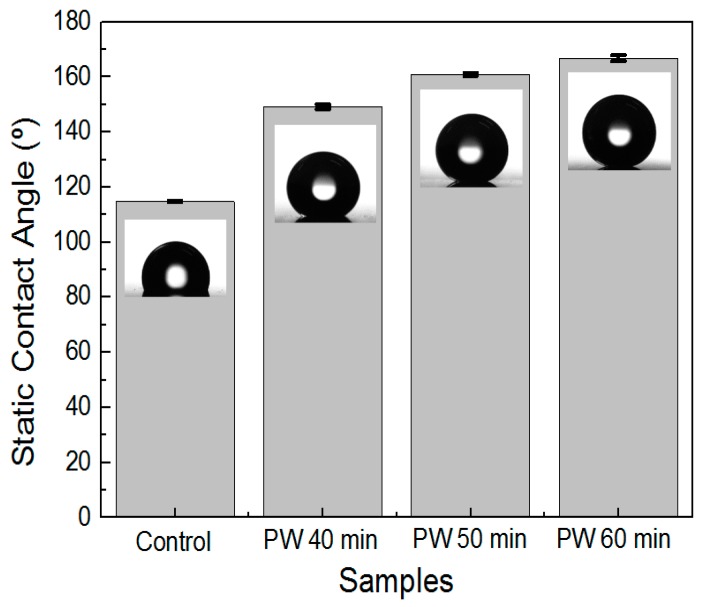
Contact angle measurements on the AAO fabricated with the HA → PW → MA process with different PW times and coated with FTDS.

Figure 4 and Figure 5 show the contact angle measurement on the hierarchical AAO nanostructures fabricated under the multistep anodization process with PW and coated with FDTS (surface energy: 5 mJ/m^2^). It is evident that for all anodization processes and PW durations, the contact angle values of the samples were increased compared to that of an untreated aluminum alloy surface.

Figure 4 shows the contact angle measurement of AAO that underwent the MA → PW → HA process with different PW times. In the case of the MA → PW → HA process, it was found that a hierarchical nanostructure was most significantly obtainable with a PW time of 40 min (Figure 2d), and this sample exhibited superhydrophobicity. The water droplets formed an almost spherical shape on the AAO surface, with an average CA value of 162.0 ± 2.04°. When the PW time was 50 and 60 min, the CA values were 142.1 ± 0.55° and 126.1 ± 0.27°, respectively.

Surface energy and surface roughness are the dominant factors in deciding the hydrophobic properties of a superhydrophobic surface. Thus, the lower the surface energy is, the higher the hydrophobicity will be. However, the FDTS (surface energy: 6.7 mJ/m^2^) can only provide an apparent contact angle of 119° [35]. Therefore, enhancement of the surface roughness is required to obtain the highest hydrophobicity [36,37]. Considering the Cassie–Baxter wetting state [38], where air is trapped within pores or between solid and liquid interfaces, the static contact angle (θ) can be described by:

(3) $$\cos\theta =r_{f}f_{SL}cos\theta_{0}-1+f_{SL}$$

(4) $$f_{SL}=1-\frac{2\pi }{\sqrt{3}}\frac{r^{2}}{a^{2}}$$

where θ_0_ is Young’s contact angle on a smooth surface, $f_{SL}$ is the fraction of the solid–liquid wet surface (defined as the ratio of the actual area of liquid–solid contact to the projected area), and $r_{f}$ is the roughness of the wetted solid surface (where unity denotes a chemically homogeneous surface). From Equations (3) and (4), it can be found that smaller values of $f_{SL}$ result in greater contact angles on a superhydrophobic surface. The pillar structures of AAO can effectively enhance $r_{f}$ and reduce $f_{SL}$. The structural features (pore diameter, interpore distance, and solid fraction) were summarized in Table 4. The higher solid fraction indicated that the contact angle is a greater value in each process (MA → PW → HA and HA → PW → MA). That is, the AAO morphology plays an important role in determining the characteristics of a hydrophobic surface. This demonstrates that a hybrid structure with a pillar structure can lower the solid fraction and thus significantly enhance the superhydrophobicity.

Figure 5 shows the contact angle measurement of AAO that underwent the HA → PW → MA process with different PW times. In this case, increasing the PW time increased the effectiveness of the pillar nanostructures (Figure 3d–f). The average CA value for a PW time of 40 min was 149.2 ± 0.78°. As the PW time reached 50 min, the sample exhibited superhydrophobicity. The water droplets formed an almost spherical shape on the AAO surface, with an average CA value of 161.7 ± 0.56°. When the PW time was increased to 60 min, the water droplets attained an even more spherical shape on the AAO surface, with CA values as high as 166.8 ± 1.09°. These CA values reveal that with given low surface energy, the surface wettability of both hierarchical and pillar AAO is affected by the surface shape and roughness. Therefore, the above results demonstrate that the pillar hierarchical structure of AAO with a less solid faction displayed the largest superhydrophobic surface.

## 4. Conclusions

This paper demonstrated the successful fabrication of both hierarchical and pillar-like AAO nanostructures on 5052 aluminum alloy using multistep anodization and a PW process. In particular, different anodization voltages were applied alternately to fabricate the hierarchical structures. Typically, the pillar structure does not show a unified AAO produced under the MA → PW → HA conditions, due to difficulty in producing more uniform pillar-like nanostructures than pure aluminum. However, a uniform pillar nanostructure under the HA → PW → MA condition was successfully demonstrated.

Pillar AAO nanostructures created through multistep anodization significantly enhanced the surface roughness. After a hydrophobic coating, the tip-like structure of AAO exhibited superior superhydrophobic efficiency with a high contact angle (<166°). Through a process for adjusting the surface shape, more air can be trapped inside the nanoscale pore structure, resulting in enhanced superhydrophobic properties. The hybrid nanostructures such as pillars created by anodization not only improve hydrophobicity, but are also inexpensive and easy to produce, which will greatly benefit industrial and engineering applications.

## Figures and Tables

**Table 1:** Fabrication process conditions for multistep anodization. HA: hard anodization, MA: mild anodization, PW: pore widening.

Sample	First Anodization	AAO Removal	Second Anodization		Pore Widening	Third Anodization	
	Time (hour)	Time (hour)	Step	Time (min.)	Time (min.)	Step	Time (min.)
A	6	10	MA	30	40	HA	0.5
B	6	10	MA	30	50	HA	0.5
C	6	10	MA	30	60	HA	0.5
D	6	10	HA	0.5	40	MA	30
E	6	10	HA	0.5	50	MA	30
F	6	10	HA	0.5	60	MA	30

**Table 2:** Characterization of the AAO structures on 5052 aluminum alloy.

Sample	Second Anodization Step	Pore Widening	Third Anodization Step	Second Anodization Region with PW		Third Anodization Region	
	Type	Time (min.)	Type	D_p_ (nm)	D_int_ (nm)	D_p_ (nm)	D_int_ (nm)
A	MA	40	HA	85 ± 1.9	100 ± 1.7	30 ± 5.2	115 ± 9.5
B	MA	50	HA	None	None	31 ± 2.2	182 ± 22
C	MA	60	HA	None	None	33 ± 2.5	219 ± 38
D	HA	40	MA	95 ± 4.5	137 ± 7.3	19 ± 1.5	94 ± 1.8
E	HA	50	MA	134 ± 3.2	185 ± 78	18 ± 1.8	90 ± 6.3
F	HA	60	MA	None	None	135 ± 4.3	182 ± 7.3
						16 ± 1.3	99 ± 7.3

**Table 3:** Average contact angle values after FDTS coating. FDTS: 1H, 1H, 2H, 2H-perfluorodecyltrichlorosilane.

Sample	Contact Angle (Distilled Water) (°)	Deviation (°)
Control	114.8	0.31
A	162.0	2.04
B	142.1	0.55
C	126.1	0.27
D	149.2	0.78
E	161.7	0.56
F	166.8	1.09

Multistep anodization process with intermediate PW; Control: Electropolished Al 5052; Sample A: MA → PW (40 min) → HA; Sample B: MA → PW (50 min) → HA; Sample C: MA → PW (60 min) → HA; Sample D: HA → PW (40 min) → MA; Sample E: HA → PW (50 min) → MA; Sample F: HA → PW (60 min) → MA.

**Table 4:** The structural characteristics of the FDTS-coated AAO nanostructures.

Sample	Pore Diameter (nm)	Interpore Distance (nm)	Solid Fraction
A	85 ± 1.9	100 ± 1.7	0.348 ± 0.007
B	31 ± 2.2	182 ± 22	0.973 ± 0.003
C	33 ± 2.5	219 ± 38	0.978 ± 0.004
D	95 ± 4.5	137 ± 7.3	0.564 ± 0.005
E	134 ± 3.2	185 ± 78	0.524 ± 0.997
F	135 ± 4.3	182 ± 7.3	0.496 ± 0.072

## Abbreviations

AAO: anodic aluminum oxide; D_p_: pore diameter; D_int_: interpore distance; PW: pore-widening; MA: mild anodization; HA: hard anodization.
